# Use of Mobile Clinical Decision Support Software by Junior Doctors at a UK Teaching Hospital: Identification and Evaluation of Barriers to Engagement

**DOI:** 10.2196/mhealth.4388

**Published:** 2015-08-13

**Authors:** Rakesh Patel, William Green, Muhammad Waseem Shahzad, Chris Larkin

**Affiliations:** ^1^ University of Leicester Department of Medical & Social Care Education Leicester United Kingdom; ^2^ University Hospitals of Leicester NHS Trust Leicester United Kingdom; ^3^ University of Leicester School of Management Leicester United Kingdom; ^4^ Health Education East Midlands Ruddington United Kingdom

**Keywords:** clinical decision support systems, health care technology, human-centered computing, medical education, patient safety, ubiquitous and mobile computing

## Abstract

**Background:**

Clinical decision support (CDS) tools improve clinical diagnostic decision making and patient safety. The availability of CDS to health care professionals has grown in line with the increased prevalence of apps and smart mobile devices. Despite these benefits, patients may have safety concerns about the use of mobile devices around medical equipment.

**Objective:**

This research explored the engagement of junior doctors (JDs) with CDS and the perceptions of patients about their use. There were three objectives for this research: (1) to measure the actual usage of CDS tools on mobile devices (mCDS) by JDs, (2) to explore the perceptions of JDs about the drivers and barriers to using mCDS, and (3) to explore the perceptions of patients about the use of mCDS.

**Methods:**

This study used a mixed-methods approach to study the engagement of JDs with CDS accessed through mobile devices. Usage data were collected on the number of interactions by JDs with mCDS. The perceived drivers and barriers for JDs to using CDS were then explored by interviews. Finally, these findings were contrasted with the perception of patients about the use of mCDS by JDs.

**Results:**

Nine of the 16 JDs made a total of 142 recorded interactions with the mCDS over a 4-month period. Only 27 of the 114 interactions (24%) that could be categorized as on-shift or off-shift occurred on-shift. Eight individual, institutional, and cultural barriers to engagement emerged from interviews with the user group. In contrast to reported cautions and concerns about the impact of clinicians’ use of mobile phone on patient health and safety, patients had positive perceptions about the use of mCDS.

**Conclusions:**

Patients reported positive perceptions toward mCDS. The usage of mCDS to support clinical decision making was considered to be positive as part of everyday clinical practice. The degree of engagement was found to be limited due to a number of individual, institutional, and cultural barriers. The majority of mCDS engagement occurred outside of the workplace. Further research is required to verify these findings and assess their implications for future policy and practice.

## Introduction

### Background

Although the influence of evidence-based medicine (EBM) on health care is gaining in importance, there can be challenges for health care professionals to practice EBM at the point of care [1]. Clinical decision support (CDS) systems, defined as “information systems designed to improve clinical decision making” [2], enable health care professionals to leverage the benefits of technology and access the latest evidence to guide their clinical practice [3]. Traditional forms of CDS range from electronic patient record database systems in which clinicians can access patient details and retrieve relevant drug information, through to standalone software applications that are effectively a repository or textbook of guidelines on a given clinical topic [2]. Despite the affordances brought by these CDS systems, a number of individual, organizational, and technological barriers affected the engagement of clinicians with the technologies [4].

Smartphones enable users to perform tasks such as replying to email and accessing Internet-based resources [5]. They can increase the productivity of people in the workplace, but can also provide an additional burden and distraction. They are increasingly prevalent with over 82% of doctors reported to be using a smartphone in the workplace to facilitate their care for patients [6].

The prevalence of smartphones among those entering the workforce is high, with 92% of junior doctors (JDs) owning such a personal device [6,7]. In the United Kingdom, JDs include foundation year (FY) doctors (those in their first 2 years of training following graduation) and core trainee (CT) doctors (in years 3-5 following graduation). This prevalence is increasing and parallels other trends such as the growth in health care-related apps, with over 10,000 now available [8]. This suggests that ownership and usage of mobile software applications among this group is already ubiquitous. Nevertheless, there is little understanding about the use of CDS on mobile phone (mCDS) devices by JDs for improving clinical care.

There are a wide range of papers describing the use of mCDS [9], yet few focus on the factors affecting engagement with mCDS by JDs. Previous studies explored the use of CDS on technology such as personal digital assistants [10] yet barriers to the use of these devices such as usability and functionalities likely relate to the outdated hardware, rather than the CDS tools per se. Although this evidence remains useful for understanding the challenges with technology acceptance among medical staff, more research about engagement with CDS following the development of smartphone devices is necessary. JDs are poor at answering their clinical questions and likewise they require significant support for finding answers on traditional CDS tools [11]. Therefore, better understanding about the usefulness of smartphones to meet their needs is required.

### Study Objective

**Figure 1:**
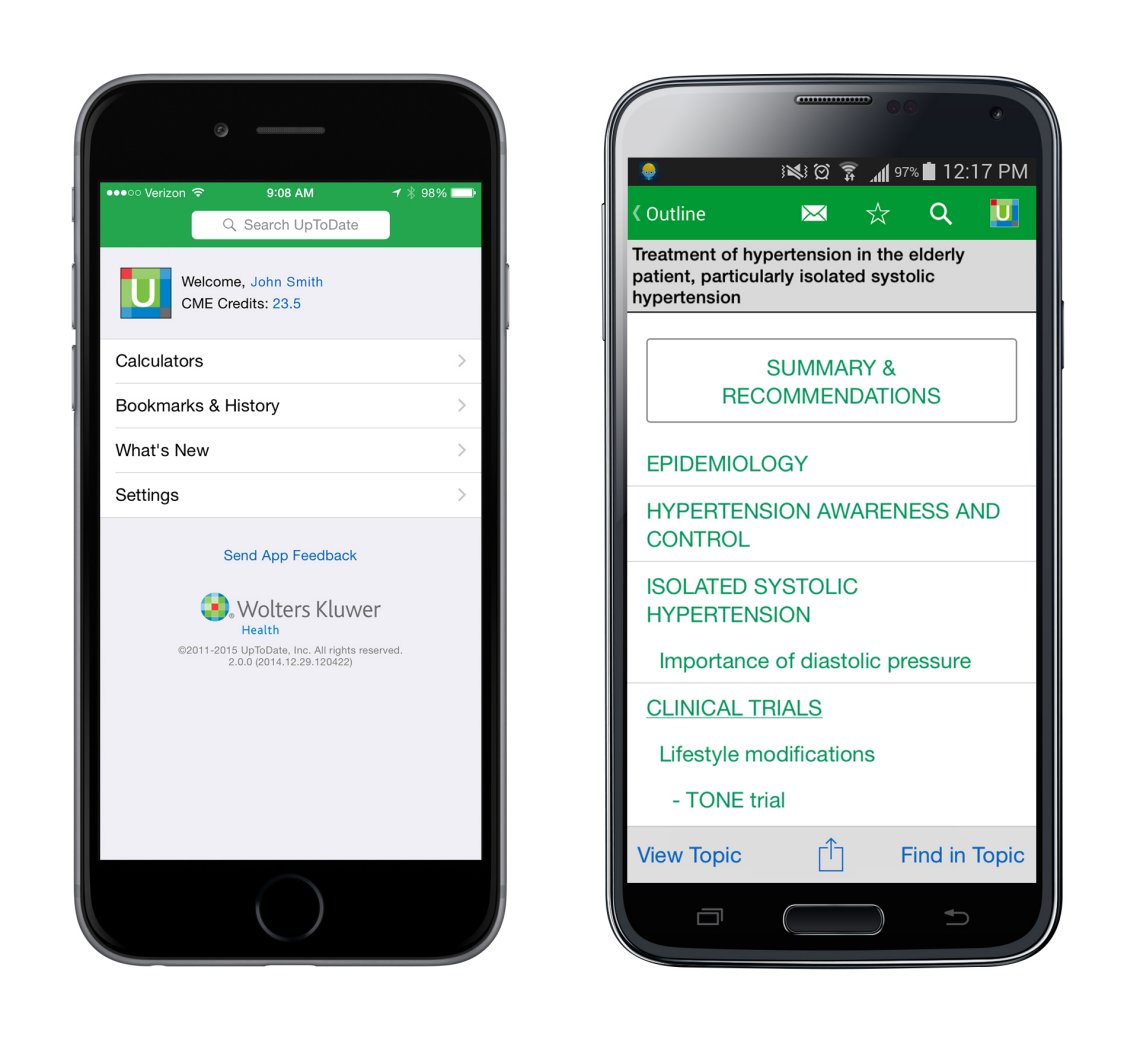
An example of a clinical decision support system on mobile phones (UpToDate) on a smartphone displaying the home page and an Android device displaying an example topic.

We aimed to explore the factors influencing JD engagement with mCDS (Figure 1) for answering clinical questions in the workplace. The objectives of this study were to quantify the usage of mCDS by JDs; to compare the perceived drivers and barriers held by individual JDs with their usage of the technology; and to triangulate these findings with patient perceptions about JDs using mCDS in the workplace.

## Methods

### Methodology

A mixed-methods approach was used for researching engagement with technology among JDs in this health care setting [12,13]. Mixed-methods approaches give researchers flexibility for exploring complex phenomena such as technology engagement, and enable data derived from multiple sources to be triangulated so that such complex phenomena can be more accurately explained.

### Context

The study was completed as part of the University Hospitals of Leicester NHS Trust’s wider clinical effectiveness (study reference number 6608E) and quality improvement program (Health Education East Midlands study reference number LEI0085), based in the East Midlands, United Kingdom. Therefore, all issues related to perceptions of surveillance and temporary behavior changes were minimized. The study was undertaken across 4 in-patient wards in a tertiary center renal unit with a total of 59 beds. All patients are admitted under specialist renal care. Consent was obtained from all JDs and patients who participated in the study and both were reminded of their right to withdraw consent at any point during the period of data collection and analysis, before dissemination of the findings. All data were anonymized to remove personally sensitive or identifiable information. At the time of the study, all medical notes at the Trust were handwritten and there were no electronic patient records. There was no electronic prescribing system; however, hospital guidelines and British National Formulary were available on the hospital intranet, accessible from any desktop computer located on all wards [14]. As this study was conducted as part of the clinical effectiveness program, JDs were not asked to stop using other CDS that they were familiar with as this could have impacted patient care.

### Sample

Sixteen JDs (FY1, FY2, and CT levels) were invited to participate in this study. These doctors were based on the renal unit as part of their individual training rotations across a 4-month period between August and November 2013. The doctors were provided with personal access to CDS technology (UpToDate) [15] on their mobile phone or equivalent device at the start of the rotation.

### Data Collection

#### Overview

Data were collected from the following 3 main sources:

1. JDs usage with mCDS
2. JDs perceptions toward mCDS
3. Patients’ perceptions toward mCDS.

#### Usage of mCDS

The usage statistics from JDs accessing the technology were collected to measure the quantity of mCDS use. An interaction was classified as a text search conducted by an individual for a specific query (eg, tacrolimus, transplant rejection, or heparin infusion). Other data were logged by the system, but they do not provide any insight into participant system usage. This includes, for example, system synchronization to receive software updates, Internet protocol address changes, and error logs.

Any interactions made by JDs while online, in this case text searches, were transmitted to a central server in real time. Usage data from offline mode were transferred when individuals next used the software online. All data were organized according to the time of the interaction to investigate periods of high and low engagement. The usage data were triangulated with the on-shift commitments of JDs to further contextualize the nature of usage with mCDS.

#### JD Perceptions Toward mCDS

To investigate the perceptions of JDs toward mCDS technology, semistructured interviews were conducted with JDs on 2 occasions during the rotation. The first interviews exploring factors affecting mCDS use were conducted after 2 months so JDs had enough time to settle into the workplace and develop ways of integrating the technology into their daily work. The second interviews were conducted after a further 2 months at the end of the job, to evaluate the main factors that promoted or prevented use of mCDS during the rotation. Prompts during the interviews included JDs’ perceived usage, their perceived usefulness and usability of the mCDS, and perceptions about acceptability of use in the workplace in front of patients.

#### Patient Perceptions Toward Engagement With mCDS

Feedback from patients about their perceptions of JDs using mCDS in the workplace was collected using semistructured interviews. Only patients who had observed first-hand JDs using mCDS on the ward were invited to share their reflections. Only members of the local area Kidney Patient Association could be approached. Patients were asked to describe their recollections of a doctor’s mCDS usage at the bedside, on the ward, or in other instances when they observed interactions with the technology.

### Data Analysis

#### Overview

Quantitative and qualitative analyses were conducted on the data collected using 3 methods. All interviews were transcribed verbatim.

#### Usage of mCDS

Interactions with mCDS were divided into on-shift and out-of-hours interactions by cross-referencing the time of interaction with available on-shift data. On-shift data were not available for 2 JDs.

#### JD Perceptions Toward mCDS

A framework analysis of emergent themes based on the integrative model of technology acceptance among professionals [16] was completed on the qualitative data collected from JDs. The themes are outlined in Table 1.

**Table 1:** Outline of the themes included in the integrative model of technology acceptance among professionals [16], which were used in the framework analyses of the interviews.

Framework themes	Definition
Personal innovativeness in information technology	The willingness of an individual to try out any new information technology.
Result demonstrability	The extent to which the tangible results of using an innovation can be observable and communicable.
Image	The extent to which use of an innovation is perceived as enhancing one’s own image or status.
Subjective norm	The perception that other people considered important by the person think that he or she should perform the behavior.
Perceived behavioral control	The perception of internal and external resource constraints on performing the behavior.
Perceived ease of use	The extent to which a person believes that using the system will be free of effort.
Perceived usefulness	The extent to which a person believes that using the system will improve his or her job performance.
Behavioral intention	A person’s subjective probability to perform a specified behavior.

The raw data were explored so that codes were applied to phrases which aligned to components of the model. Any code or theme that did not align with a component in the model was identified as an emergent theme and organized into a new component. All codes within components and existing or new themes were triangulated with findings from the other analysis to explain mCDS engagement among JDs.

#### Patient Perceptions Toward Engagement With mCDS

A thematic analysis was completed on the qualitative data collected from patients. This approach was chosen because there was no expectation that the process of coding would fit the data into a pre-existing model or frame. This inductive or data-driven approach ensured all themes were rooted in the raw data where the focus of inquiry was patients’ perceptions, feelings, and experience of JDs’ mCDS usage. In particular, the relationship between patients’ subjective experience of mCDS usage by JDs and their confidence with the problem-solving or decision-making skills of the individual in question was explored. Furthermore, themes that identified a relationship between JD mCDS usage at the point of care and patients subjective experience about safe or effective care on the ward were also explored. Finally, the themes identified by patients and JDs about mCDS were compared and contrasted.

## Results

### Usage of mCDS

A total of 142 mCDS interactions across 16 JDs were recorded during the 4-month study period (Table 2).

Five JDs made 14 or more recorded interactions. This equates to 90.1% of all observed interactions (n=128). Seven JDs did not use the mCDS software. The JD who interacted the most made 43 interactions, equating to 11 interactions/month. The mean number of interactions across the JDs who used mCDS (excluding those who did not interact at all) was 4 interactions/month. The 2 JDs who had the most interactions (43 and 36) were both FY1. These 2 JDs had nearly twice as many interactions as the JD with the next highest number of interactions (20). While this is potentially interesting, no further statistical analysis has been performed due to the small sample across the 3 occupational-grade groups.

Data from 14 of the 16 JDs were available to establish whether mCDS usage was conducted while on-shift or not. Of the 113 accountable interactions, 27 interactions were recorded while JDs were on-shift, and 86 interactions were recorded when JDs were off-shift. This suggests that a greater proportion of interactions are conducted off-shift. Unfortunately, the interactions for JD 4 and JD 5 could not be categorized as either on-shift or off-shift.

**Table 2:** Recorded interactions with clinical decision support on mobile phones among junior doctors.

Junior doctor (JD)	JD grade	Total interactions (n)	On-shift interactions (n)	Out-of-hours interactions (n)	Interview conducted?
(Yes/No)
1	FY1	43	18	25	No
2	FY1	36	5	31	No
3	CT	20	1	19	Yes
4	CT	15	N/A	N/A	Yes
5	FY2	14	N/A	N/A	Yes
6	FY2	6	1	5	Yes
7	CT	4	0	4	Yes
8	FY1	2	2	0	Yes
9	FY2	2	0	2	Yes
10	FY1	0	0	0	Yes
11	FY2	0	0	0	Yes
12	FY1	0	0	0	Yes
13	FY1	0	0	0	Yes
14	FY1	0	0	0	No
15	FY2	0	0	0	No
16	FY1	0	0	0	Yes
Total	16	142	27	86	Yes = 12

### JD Perceptions Toward mCDS

#### Overview

Twelve JDs completed a semistructured interview exploring their perceptions about using mCDS (Table 2). Four JDs were not available for interview due to their availability. The main themes that explain the engagement of JDs with mCDS based on the framework analyses of the integrated model of technology acceptance among professionals [16] (Table 3) relate to personal innovativeness, and the impression given by the JDs when using mCDS to others around them (image and subjective norm), perceived ease of use, and perceived usefulness. Eight barriers or reasons for nonengagement with the mCDS emerged from these themes. They are categorized as being individual, institutional, or cultural (Table 4).

**Table 3:** Summary of the framework analysis of junior doctor perceptions of clinical decision support on mobile phones (themes that did not emerge have been removed).^a^

Positive or negative perception	Framework theme	Personal innovativeness in information technology	Image	Perceived ease of use	Perceived usefulness	Subjective norm
Positive perceptions toward clinical decision support on mobile phones (mCDS) software	Number of times theme emerged	0	0	2	3	0
	Example comment	—	—	*It is fantastic to have it available...on your phone as well, that's brilliant.*	*I had downloaded it on my phone and I found it really helpful.*	—
Negative perceptions towards mCDS software	Number of times theme emerged	1	1	1	0	1
	Example comment	*I need to sign up for an Athens account and I haven't really done that either, purely because we've been updated with so many passwords and usernames. I thought this is one too many, I can't cope.*	*My major issue with it in terms of using it at work is still the acceptability of using mobile phones in a ward environment where everyone assumes that you are doing a million and one other things but certainly not looking up educational materials. I'm sure the patient still thinks that you are calling and arranging your social life.*	*If we are talking about BNF [British National Formulary app] I only use my phone, I don't use anything else because it's faster, it's easy access. But, this [mobile CDS software] is so much worse, it is killing me.*	—	See image comment

^a^This is based on the integrative model of technology acceptance among professionals [16]. Themes that emerged to explain the behavior of junior doctors (JDs) with clinical decision support on mobile phones (mCDS) related to perceptions around the ease of using mCDS and the perceived usefulness of mCDS for working as a JD, personal initiative, and capability for using technology in general, as well as the impression given by the JDs when using mCDS to others around them and the subjective norm. Eight individual, institutional, and cultural barriers were identified from these themes.

**Table 4:** Individual, institutional, and cultural perceptions to explain the engagement with clinical decision support on mobile phones. The 3 categories emerged from 8 subthemes.

Perceptions	Theme	Example
Individual	Usability: Small screen hard to read	*I don't particularly use it on my phone. I sometimes do, but it tends to be not necessarily at the bedside or on the ward because it's quite hard to look at on the phone. [JD 7, 4 interactions]*
		*I use it on the computer a lot of the time...rather than using it on my phone, just because we have quite a few computers on the ward and it is just easier, bigger screen. [JD 13, no interactions]*
		*I prefer the computer you know…the mobile is small. I try to use it from the computer, it's much more comfortable. [JD 3, 20 interactions]*
	Fit-for-purpose: clinical decision support on mobile phones (mCDS) preferred as out-of-hours learning resource	*I use it more for MRCP [Membership of the Royal College of Physicians diploma] revision I think rather than actually looking things up for work. I've only used it maybe once or twice at work for work reasons. [JD 6, 6 interactions]*
	Perceived lack of time	*[A barrier is] physically having time allocated to be able to look through these different resources. [JD 10, no interactions]*
	Existing resources equally effective	*I generally ask someone...because there's generally a lot of people around, or use the hospital guidelines, they are really quite good. [JD 11, no interactions]*
		*[I use] mostly BNF online, BNF paper copy. I’ve used up-to-date a few times. [Interviewer: On your own phone?]* *No. On the computer. And also asking the pharmacist questions, and asking the seniors questions and the...actual Internet of the hospital. [JD 7, 4 interactions]*
Institutional	Information conflicts with local/national guidelines	*[A barrier is] knowing that most of what you are researching is going to be overruled by local guidance anyway, so it doesn't actually tie in with what your local trust policy is saying. [JD 10, no interactions]*
		*The other thing I've found with [the mCDS] was that...it's not NICE Guidelines...and it's not, not always Trust guidelines in terms of, sort of, management and prescribing...There's a Trust policy on those sorts of things...If one deviates from the other, obviously you're going to go with the Trust policies, not [mCDS]. [JD 12, no interactions]*
		*The problem is though...when you are working in a big Trust really, you should be following clinical guidelines that are available. [JD 8, 2 interactions]*
	Lack of support	*I think I need to sign up for an Athens account and I haven’t really done that either, purely because we’ve been updated with so many passwords and usernames. I thought this is one too many, I can’t cope. [JD 12, no interactions]*
		*They said before our rotation they will send [information about the mCDS introduction] meal as well. [Interviewer: Really?] Yeah, and then they kept asking us, “Did you went the [mCDS introduction] meal?” [sic] I was like, “We did not receive any email regarding the [mCDS introduction] meal.” [JD 9, 2 interactions]*
	Lack of supporting technological infrastructure	*The [Hospital] is not blessed with [Wi-Fi] signal, so that makes it very difficult to try and access things on your phone. [JD 12, no interactions]*
Cultural	Prevailing cultural norm surrounding technology discourages use of mobile devices at bedside	*I think my major issue with it in terms of using it at work is still the acceptability of using mobile phones in a ward environment where everyone assumes that you are doing a million and one other things but certainly not looking up educational materials...I'm sure the patient still thinks that you are calling and arranging your social life and so I just don't like that gap in terms of people accepting that you can be using or texting or being on Facebook or something. That has limited how much I've used it in the workplace, and I will really miss not having it soon on my mobile device. [JD 4, 15 interactions]*

#### Individual Barriers

JDs preferred using the desktop-based CDS to the mCDS where reading information on a small screen in certain clinical contexts was perceived as challenging. JDs also preferred using mCDS as a learning resource in their own time rather than as a tool exclusively to aid them at the bedside in the workplace. Furthermore, JDs suggested difficulty integrating mCDS into their pattern of work, which was conditioned through previous jobs where mCDS was not available. As a consequence, JDs had difficulties integrating mCDS into their role in the workplace and so sought instead to use alternative and more established sources of support when presented with a clinical question. On direct probing of these alternative sources, at least seven were cited by JDs within interviews (Table 5). It is particularly surprising that colleagues were not consulted for a second opinion more often as a CDS resource, which is in contrast to previous findings [17].

**Table 5:** Alternative sources of clinical decision support reported by junior doctors in this study.

Resource	Number of junior doctors referring to resource
UpToDate desktop [18]	7
British National Formulary Desktop [19]	6
British National Formulary book [14]	3
British National Formulary App [20]	2
Colleagues as a “second opinion” (seniors/pharmacists)	2
Local clinical guidelines	1
Academic journals	1

#### Institutional Barriers

JDs confirmed that inconsistencies between recommended and expected clinical practice presented challenges to the adoption of mCDS guidance. A regular dilemma for JDs was choosing between suggestions given by mCDS and receiving instruction from alternative, traditionally “trusted” sources. This dilemma was more challenging when instruction varied across sources such as local, mCDS, and national guidance (eg, National Institute for Health and Care Excellence clinical guidelines) [21]. JDs gave up using mCDS in these situations, defaulting to resources perceived as being more accessible such as desktop computers.

The motivation for JDs to persist with using mCDS against this backdrop was challenged, especially in the face of other barriers such as reported usability and accessibility issues. Although JDs cited usability and accessibility as a factor for deterring the use of mCDS, all were provided with personal subscriptions for the software to enable access with minimal effort and all participated in an induction session where mCDS was carefully introduced to them. Irrespective of these specific accessibility issues, JDs also cited the general lack of information communications technology infrastructure as a barrier to active mCDS use.

#### Cultural Barriers

Some of the JDs explicitly expressed belief that the use of mCDS in direct view of patients would be perceived as being unprofessional. They, therefore, chose not to use devices in plain view. This concern was also raised in relation to senior colleagues considering JDs’ use of their mobile device in front of patients or on the ward as being unprofessional. This dissuaded JDs from using mCDS at and away from the bedside. Paradoxically, other JDs acknowledged the opportunity for using mCDS positively and so used mCDS, as they felt it appropriate and had less concern for the negative views of others.

### Patient Perceptions Toward mCDS

Four kidney patients were interviewed as part of the study. Patients interviewed were all members of the local area Kidney Patient Association. At the time of the study only 4 of their members were on the ward. The study was not permitted to approach other patients due to confidentiality reasons.

All patients were in favor of using technologies such as mCDS to better inform clinical diagnostic decision making. Furthermore, all the patients were comfortable with JDs using the mCDS at the bedside as part of the consultation process if appropriate to the delivery of care.

I don’t mind, I’m quite happy with that. There’s so many drugs and so many side effects and whatever, I want them to be as informed as possible, please.

Patient 3

Patients acknowledged the complexity of their medical condition, which comprised multiple long-term conditions. For the patients, deciphering the condition that contributed to their presenting problem was not obvious. Furthermore, patients believed that prescribing medication and avoiding drug interactions required JDs in the absence of clinical experience to seek support from a variety of sources such as mCDS.

Patients believed accessing mCDS was equivalent to asking senior clinicians for the answer when making a clinical decision.

When the doctor says to you, “I’ve got to go and consult a senior,” we exactly know what they’re going to do. It’s the same thing. Otherwise they say to you, “I have to go and look it up.”

Patient 1

Patients also felt reassured that JDs sought to use mCDS in their clinical diagnostic decision making, rather than make decisions without some form of support in a setting of uncertainty. Furthermore, patients believed that the comfort with using mCDS in the clinical diagnostic decision-making process communicated something positive about the confidence and competence of the health care professionals.

...a GP [General Practitioner] that was looking after me, he was ready to say, “I want to check on one or two things,” and he would pull them up on his PC. You can’t know everything. I think it’s reassuring to know that your physician isn’t pompous enough that it stops them from genning-up [revising] on something that they’re uncertain about or totally ignorant, maybe. So I’d rather they do that...I think it’s reasonable to use the tools of the day, and [mCDS is] one of them.

Patient 4

Patients stressed the importance of JDs explaining their intention for using mCDS prior to doing so. While patients were supportive of mCDS, all felt that a brief explanation of the rationale for using mCDS in the consultation was important to prevent any misappropriation of their behavior, such as handling of devices that could be seen as an intention to use them for nonwork-related purposes.

If the doctor does have to use a mobile while he’s with the patient, he has to just tell the patient, “Listen, I’m looking up a certain drug, I want to see what it says about it,” and the patient will perfectly understand that...As long as they explain it, that’s fine.

Patient 1

If they say, “I’m just going to use this to just check on this, because it could have side effects or it could have something, so, do you mind if I just look at it now?” That’s all they need to do, isn’t it?”

Patient 3

## Discussion

### Descriptive Findings

This research explored the factors influencing JDs engagement with mCDS for answering clinical questions when in the workplace; in addition, we compared the perceived drivers and barriers held by individual JDs with their usage of the technology and considered these findings in relation to patient perceptions about JDs using mCDS in the workplace. Surprisingly, of the 16 participants who had the opportunity to utilize the mCDS through the free, personal subscription, no interactions were recorded for 7 JDs. As many as 128 (90.1%) of the interactions were recorded by 5 of the 16 JDs, with a range from 14 to 43 interactions/JD, indicating a large individual variance. The remaining 14 interactions (10%) were recorded by the remaining 4 JDs. Of the 9 JDs who did interact with CDS, 4 interactions were recorded on average/month. An interesting finding is that the majority (n=86) of the interactions were conducted out-of-hours.

mCDS is more accessible to the end user at the bedside to make clinical decisions, compared with equivalent systems available on desktop computers. JDs who lack experience are more likely to seek information to support clinical diagnostic decision making compared with clinicians who are more likely to use experience as a driver for decision making [17,22]. The usage of mCDS was initially considered low by the research team (4 interactions/month for those who did engage). However, the engagement of JDs is more than other research which concluded that bedside use of CDS was, “feasible and useful in addressing unresolved clinical questions” [17]. For example, Phua et al [17] reported 157 searches by 27 doctors (5 consultants, 2 associate consultants, 4 registrars, 13 medical officers, and 3 house officers) between their study period from September to November [17]. Nonetheless, these figures do seem to be low given the number of clinical decisions made in practice. The barriers identified in this and previous studies provide explanations for this low engagement which should be addressed in future research.

At first glance, the attractiveness to using mCDS for JDs may appear to be when physical presence in the form of senior support is lacking; however, this study did not confirm this assumption. In the absence of previous studies exploring the use of mCDS among JDs, possible reasons for reduced mCDS use while on-call may include the increased availability of senior advice or the perceived lack of time from volume of work. A previous study identified that JDs found lack of time as a pervasive barrier to answering their clinical questions with evidence-based support tools accessible on desktops [23].

### Junior Doctors’ Perceptions Toward mCDS

The main barriers to mCDS were anticipated following a review of conceptual models for explaining technology acceptance by health care professionals [16,24-27]. These models did not predict all the barriers identified in this study. Part of this is due to the model variables not including some of the specific characteristics unique to mCDS. Previous researchers have criticized the exclusive use of models to explain drivers or barriers for engagement with technology for this very reason [28,29]. Although existing models may explain an individual’s intention to use a given technology such as mCDS, they lack solutions to overcome context-specific barriers to engagement. This suggests that the models need to be revisited in relation to mobile-specific technology in tertiary care.

### Individual Barriers

Usability issues were a barrier to mCDS use in this study. This supports the findings from other studies in general workplace settings including health care, which have identified screen size as a particular issue for end users [30,31]. However, some studies report that doctors prefer smaller devices with better form function over a larger device that would be easier to read at work [32], suggesting that usability issues are context specific and require further research in the health care setting. Alternatives to smartphone access for improving usability issues include the provision of tablets; however, other factors such as the risk of theft in the health care setting need careful consideration [33].

The extent of mCDS usage outside the workplace rather than within it has not been extensively reported because research to explore engagement of technologies has generally taken place as during work hours [17]. This observation suggests that the attractiveness of mCDS among JDs was sufficient to encourage them into accessing the resource away from the workplace, despite suggesting perceived lack of time was also an issue. Similarly, the range of alternative sources used for accessing other CDS systems suggests that it is not the perceived usefulness of CDS in general that is of concern. Previous studies in North America have indicated that desktop versions are most widely used among doctors in training [34] as well as established nephrologists [35]. Alternative sources of information are likely to be advocated in some contexts and may form the basis of individual, institutional, and cultural habits. These practices may be a barrier for the adoption of mCDS but are not necessarily problematic for patient safety and decision making. Rather, CDS should be used to supplement these practices. Clinical guidelines should be developed locally to advise how inconsistent information across sources should be resolved.

### Institutional Barriers

Although JDs cited that a conflict between local guidelines and information on commercial software prevented greater engagement with mCDS, some tools do have functionality for clinicians to edit information within the software and achieve better concordance with local guidelines [36]. Further research is necessary to examine whether alternative choices represent deviations from clinical guidelines or whether the clinical context in which JDs were immersed in required an alternative approach.

Despite an induction to the technology at the start and a group-based review in the middle of the rotation, this study supports the finding from the wider literature that a perceived lack of support is likely to inhibit technology use. A literature review of evidence for measures to support technology implementation in health care confirms a lack of appropriate training and technical support as major barriers to engagement with technology [37]. Poor information technology infrastructure for new technologies such as Wi-Fi access is already a well-reported barrier for engagement with mCDS by JDs in other parts of the United Kingdom [38].

### Cultural Barriers

The culture of the renal unit emerged in the interviews as a barrier to JD engagement with the mCDS. This relates to the use of a personal mobile device on the ward to access mCDS as being perceived by patients and senior colleagues as unprofessional or demonstrating inexperience. Conversely, some of the JDs were not concerned. Previous research has demonstrated that there can be striking differences between what individuals consider to be socially appropriate mobile phone use in particular contexts [39]. Palen et al [39] demonstrated that behavior and considerations for what is deemed to be appropriate are modified quickly following experience. Nickerson et al [40] also reported differences between what is acceptable voice and texting mobile phone use based on national culture and a user’s age.

These findings suggest 2 things for future adoption of mCDS at the bedside. First, if the usage of mCDS were normal practice for all health care professionals, these barriers would be minimal. Second, national culture and age will have an impact on what is deemed to be appropriate or not. However, given the sensitive environment of health care, further research should establish whether or not Palen et al’s [39] and Nickerson et al’s [40] findings predict what is socially appropriate for mobile device use in health care. For example, Brady et al [41] reported that mobile communication devices can be contaminated with bacteria and as such procedures have to be followed to reduce contamination or banned in some more critical hospital areas. If there were a number of publicized episodes of such contamination, the perception toward mobile device use on wards and at the bedside would soon decline.

### Patient Perceptions Toward mCDS

Despite the positive feedback from patients about the use of mCDS by participants, other research suggests that patients who observe clinicians using mCDS perceive them as being having poorer diagnostic ability and as demonstrating less professional awareness compared with clinicians who do not regularly access such technologies [42,43]. In their case [42,43], research involved simulations with undergraduate students playing the role of patients; therefore, how well these perceptions generalize to the beliefs of patients in real practice is unclear.

### Limitations

There are 4 main limitations to this study. First, the lack of data attributable to individual users who accessed CDS on desktop computers limits the true engagement of JDs with CDS to be evaluated. There is a strong case for enabling greater ease of access on desktop versions, rather than forcing individuals to login (and capture their individual user information), which risks people not using the resource in the first place. Second, no data were collected on the use of other online CDS accessed through the mobile phones. A number of online reference tools such as Medscape also provide CDS; therefore, the actual engagement with CDS in the widest sense is likely to be underreported. Third, the use of mCDS by senior colleagues was not studied despite the apparent influence of their actions on the behavior of JDs. Finally, the actions that senior colleagues expect JDs to take when they are uncertain and have unanswered clinical questions were not identified in this study, although these appear to influence the behavior of JDs. Thus, these should be the subject of future research.

### Implications

The findings of this study carry a number of implications for current practice, institutional policy, and further research. The perceived lack of time cited by JDs for using mCDS raises questions about the usage and accessibility to these technologies at medical school. The need for medical students and JDs to become more digitally literate was recognized 20 years ago [44]. Twenty years later, this study’s findings lend further support for this call. Health care professionals are now working in an age where the medical knowledge doubling time is rapidly reducing and predicted to be only 73 days by 2020 [45]. Rather than squeezing more new things into undergraduate or postgraduate curricula, developing traditional communication skills courses and re-examining the role of digital devices such as mCDS in the consultation process may seem more appropriate. Furthermore, over time and with more practice using mCDS as part of a forward-looking training program, productivity and quality of patient care could improve, resulting in benefits for patients, health care professionals, and organizations [46].

Organizations should carefully consider the reported individual, institutional, and cultural barriers before implementing new technologies such as mCDS, as they otherwise risk little or no technology adoption by health care professionals. Active training and technical support must be provided to all potential end users, with protected time set aside to target barriers such as misconceptions, and give health care professionals the best chance of engaging with the software. Technical infrastructure must be evaluated prior to adopting technology such as mCDS that requires frequent updates to ensure information is up to date. Without a reliable Internet connection, the sustainability of technology adoption may be affected detrimentally, as health care professionals may soon abandon technology that is not reliably available and is potentially out of date. Buy-in from management and senior clinicians is also likely to influence uptake from JDs and the prevailing beliefs held by clinicians about new technologies accessible on mobile devices.

JDs engaged with mCDS outside the workplace, despite the primary function of the technology being a CDS tool for answering clinical questions at the bedside. Although usability and in particular screen size was reported as a barrier for mCDS use, alternatives such as tablet computers are potentially available. Clearly, more research is necessary to better understand the feasibility of providing such devices for the ward-based setting, given the associated risks such as theft. One of the main unanswered questions where there is a paucity of evidence concerns the impact of mCDS or CDS upon patient care [38]. A multisite study [47] suggested correlation between availability of a desktop-based version of CDS and a shorter length of stay with lower mortality rates for patients. However, while the effects in small and nonteaching hospitals were strong, the benefits were not as clear in larger teaching hospitals [47]. A large-scale study in the United Kingdom is necessary to confirm the benefits and assess the nature of impact before reallocating significant resources to mandate the use of any innovative CDS systems among JDs in the National Health Service.

### Conclusions

This research explored the factors influencing JD engagement with mCDS for answering clinical questions in the workplace. The usage of mCDS to support clinical decision making was considered to be positive as part of everyday clinical practice. However, there are large differences between JDs’ usage. This is attributed to individual, institutional, and cultural barriers that must be overcome for mCDS to become a part of clinical working practice. Individual barriers to engagement include usability issues such as finding information hard to read due to the small device screen, preference to use the mCDS away from the workplace, feeling pressured to have sufficient time to engage with the mCDS, and feeling more comfortable in using more familiar sources of clinical support on the ward. Three institutional barriers were reported to mCDS engagement, namely, disagreement between information given by the mCDS and local or national guidelines, a lack of support provided to JDs by the implementation team, and poor Wi-Fi coverage at the hospital. One major cultural barrier existed, in relation to JDs’ concern for being seen to use the mobile phone while interacting with patients. Patients, contrary to JDs’ concerns, felt great enthusiasm for mCDS to inform and enhance patient safety, on the assumption that JDs would explain why a mobile phone was being used as part of doctor-patient interaction.

The study observed the implementation of mCDS into clinical use for JDs and found that engagement among the user group was low, albeit more than that of similar studies. Many of the barriers identified are relevant to the implementation of all new technologies in health care. In particular, 2 barriers (providing adequate support to JDs and changing organizational culture to encourage engagement) are of particular note as these require change at the institutional level. Two novel findings emerged from the study, namely, patients reported positive perceptions of mCDS use throughout patient interactions and the majority of user engagement with the tool occurred outside of the workplace environment.

## Abbreviations

CDS: clinical decision support
CT: core trainee
EBM: evidence-based medicine
FY: foundation year
JD: junior doctor
mCDS: clinical decision support on mobile phones
